# Complication management in hypertrophic cardiomyopathy

**DOI:** 10.1007/s10741-026-10682-8

**Published:** 2026-09-30

**Authors:** Viktoria Höller, Peter-Paul Zwetsloot, Viktoria Santner, Nora Schwegel, Sophie Luise Pötsch, Nicolas Verheyen, Michelle Michels

**Affiliations:** 1 Department of Cardiology, University Heart Center, Medical University of Graz, Graz, Austria; 2 Department of Cardiology, Thoraxcenter, Cardiovascular Institute, Erasmus MC, Dr. Molewaterplein 40, Rotterdam, 3015GD The Netherlands

**Keywords:** Hypertrophic cardiomyopathy, Complication management, Heart failure, Arrhythmia, Obstruction, Ischemia

## Abstract

Hypertrophic cardiomyopathy (HCM) is the most common inherited cardiac disorder, yet remains substantially underdiagnosed in clinical practice. Although current guidelines define HCM primarily based on left ventricular wall thickness, this approach alone does not capture the full spectrum of disease expression. Comprehensive phenotyping using multimodality imaging, combined with genetic testing and systematic family screening, allows for more accurate diagnosis and earlier detection, although its integration into routine clinical algorithms remains incomplete. Clinical outcomes and therapeutic strategies in HCM are largely driven by disease-related complications. These include heart failure, atrial fibrillation, ventricular arrhythmias, and sudden cardiac death, where risk stratification and prevention are central. While left ventricular outflow tract obstruction represents a major therapeutic target with rapidly evolving treatment options, alternative obstruction phenotypes and non-obstructive disease have received comparatively less attention. In these patients, symptoms are often determined by microvascular dysfunction, diastolic impairment, and myocardial fibrosis, contributing to myocardial ischemia and heart failure. Recent advances in targeted therapies, particularly cardiac myosin inhibitors, highlight the increasing importance of precise phenotypic characterization for treatment selection. However, their long-term impact on disease progression and clinical outcomes remains to be established. Overall, HCM should be considered a dynamic and heterogeneous disease continuum requiring individualized, phenotype-driven diagnostic and therapeutic strategies.

## Introduction

Hypertrophic cardiomyopathy (HCM) is the most common inherited cardiac disorder and a leading cause of heart failure, atrial fibrillation, ventricular arrhythmias, and sudden cardiac death (SCD) across all age groups [1]. Although contemporary management has markedly improved prognosis, clinical outcomes remain largely determined by the timely recognition and treatment of disease-related complications. With the introduction of disease-modifying therapies, particularly cardiac myosin inhibitors, precise phenotypic characterization has become increasingly important not only for diagnosis but also for therapeutic decision-making and longitudinal risk assessment.

### Epidemiology

**Fig. 1:**
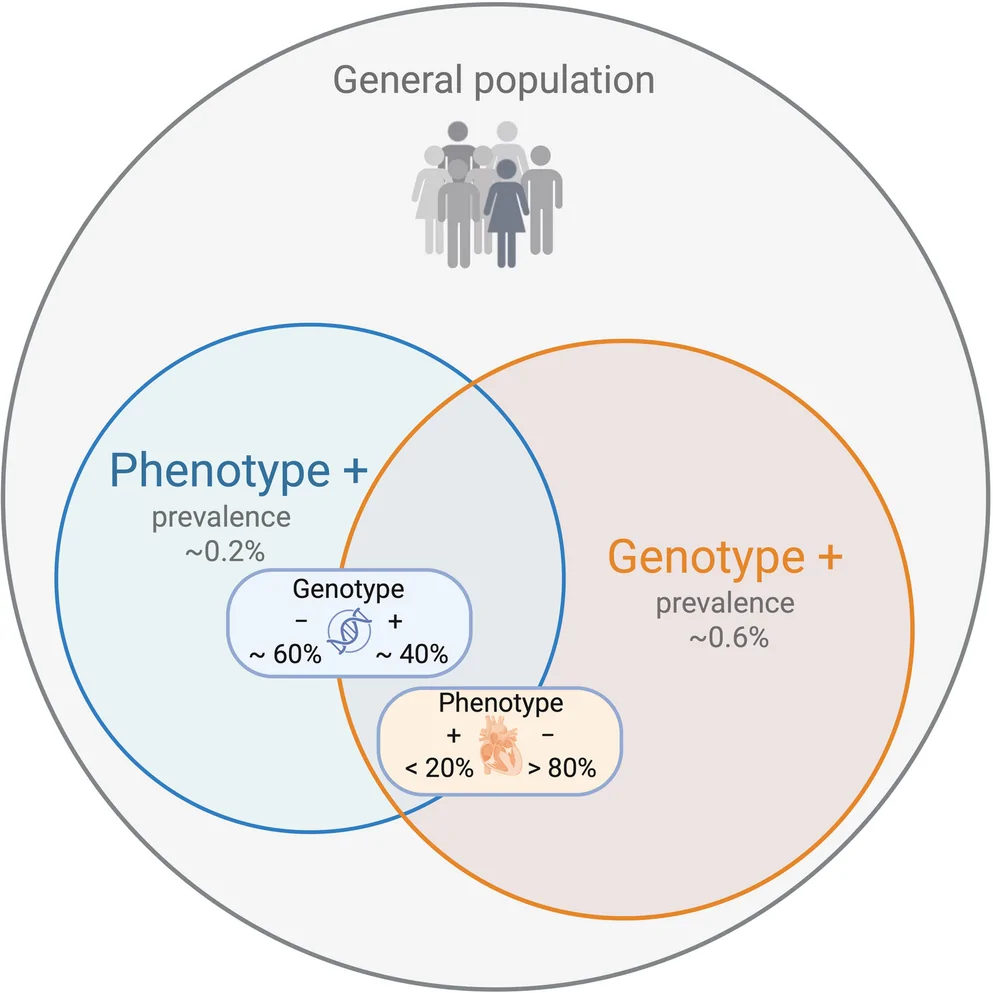
Prevalence estimates of HCM, based on phenotype and genotype screening studies [1–6]. The prevalences of the HCM phenotype and HCM-associated genotype in the general population are approximately 0.2% and 0.6%, respectively. Both groups only partly overlap, since approximately 60% of phenotype-positive individuals are genotype-negative, and more than 80% of genotype-positive individuals are phenotype-negative. Genotype +, genotype-positive; Genotype -, gene-elusive, Phenotype +, phenotype-positive; Phenotype -, Phenotype-negative. Created in BioRender. Schwegel, N. (2026) https://BioRender.com/a800hu6

Echocardiography-based phenotype screening studies in adults across various races and geographical regions consistently report a prevalence of 0.2% in the general population of whom approximately 40% are genotype posivite (G+), carrying a disease-causing sarcomeric variant (Fig. 1) [1–3]. From a genotype perspective, disease-causing sarcomeric variants occur in approximately 0.6% of the general population, but the majority of G+ individuals remain clinically silent: the reported median penetrance of disease-causing sarcomeric gene variants does not exceed 20% in the general population, and may rise to 50% only in G+ relatives of index patients [4–6].

Contrasting screening studies, the prevalence of clinically diagnosed HCM in a population wide analysis of the United States was only 1:3195, indicating dramatic underdiagnosis of HCM in real-world settings [7]. Yet, an overall increase in incident HCM has been noted over the last decades. This is driven by more gene-elusive (G-) patients diagnosed at an advanced age who are characterized by a mild phenotype but a higher proportion of left ventricular outflow tract obstruction (LVOTO) [8, 9]. This translates to women being diagnosed at higher age and more advanced disease stage [10–12].

Up to 50% of patients with HCM remain a- or oligosymptomatic, with a life expectancy comparable to patients without HCM [13]. The remainder experience symptomatic disease due to HCM-related complications, and normal life expectancy can only be achieved with their adequate management [14]. HCM-related complications include LVOTO (prevalence approximately two-thirds in referral center populations [15]) or mid-cavitary obstruction (prevalence unknown), atrial fibrillation (AF) (20% to 25% [1, 16, 17]), left ventricular (LV) systolic dysfunction (8% lifetime risk [18]), and SCD (0.7% lifetime risk [19]). Among specific treatments, the highest impact on life prolongation has been attributed to primary prevention implantable cardioverter-defibrillators (ICDs) and septal reduction therapies (SRT) for the treatment of LVOTO, while the long-term effect of cardiac myosin inhibitors on complications and mortality is yet to be defined. In a contemporary cohort from a large referral center, HCM-related mortality was 0.5%/year [20, 20]. Of note, women are diagnosed at higher age and more advanced disease stage than men, translating partly into higher risks of specific HCM-related complications [10–12]. In a recent meta-analysis comprising 44,677 HCM patients, women had a higher proportion of advanced heart failure with New York Heart Association (NYHA) functional class III/IV, and LVOTO. Women exhibited a higher risk of all-cause mortality and heart failure hospitalizations, while there was no significant difference regarding the risk of SCD [21].

### Diagnosis

Current recommendations from the European Society of Cardiology (ESC) and the American Heart Association (AHA) define HCM by a maximal end-diastolic wall thickness ≥ 15 mm in one or more myocardial segments in the absence of abnormal loading conditions, with lower thresholds ≥ 13 mm applicable in first-degree relatives of index patients and G+ individuals [22, 23]. HCM is more often diagnosed in men than in women most likely because women are on average smaller and have thinner myocardial walls than men, while recommended diagnostic wall thickness cut-offs for HCM are not adjusted for body surface area [22].

Recent studies further challenge the universality of thickness-based criteria by demonstrating phenotype-specific limitations. In apical HCM, conventional absolute thresholds perform poorly; Hughes et al. showed that indexed segmental apical wall-thickness criteria significantly improve diagnostic accuracy compared with traditional approaches [24]. These findings support a broader conceptual shift: HCM is not a uniform hypertrophic process but a heterogeneous disease with region-specific and genotype-dependent expression. Accordingly, a purely septal or maximal thickness-based definition is unlikely to capture the full disease spectrum. The limitations of thickness-based diagnosis become even more apparent in early disease and family screening. While current guidelines recommend serial evaluation of at-risk relatives using imaging [22, 23], emerging data indicate that structural hypertrophy is a relatively late manifestation. Cardiovascular magnetic resonance (CMR) feature-tracking studies have demonstrated impaired myocardial deformation in sarcomere mutation carriers without overt left ventricular hypertrophy (LVH), suggesting a detectable pre-hypertrophic stage [25]. Similarly, advanced CMR phenotyping, including tissue characterization, may identify G+ individuals before classical diagnostic criteria are met [26]. These findings imply that reliance on wall-thickness thresholds alone may delay diagnosis in genetically affected individuals. On the other hand labelling individuals based solely on borderline hypertrophy (e.g., 13 mm) without additional phenotypic or genetic evidence may not reflect true disease biology, as the penetrance and clinical significance might be lower than expected in G+ individuals [27].

Speckle tracking is an inexpensive and easily accessible technique, and may serve well as an indicator of myocardial fibrosis in HCM. In G+ individuals without overt hypertrophy, reduced left atrial reservoir strain and subtle abnormalities in transmitral flow and left ventricular global longitudinal strain (LV GLS) have been described, although their ability to predict subsequent phenotype development remains unproven [28]. Even though LV GLS is correlated with left ventricular ejection fraction (LVEF), N-terminal pro-B-type natriuretic peptide (NT-proBNP) and troponin T as markers of disease severity, its role as a genotype specific marker remains unclear [29, 30].

### Genetics

The genetic architecture of HCM is now understood to be more complex than the classic monogenic model. The ESC and AHA guidelines support genetic testing in clinically affected probands, primarily to identify a pathogenic or likely pathogenic variant that can enable cascade testing and family surveillance. At the same time, both guidelines acknowledge incomplete penetrance and variable expressivity noting that pathogenic variants are identified in roughly 30% of sporadic and 60% of familial cases [22, 23]. Emerging literature has moved the field decisively away from the notion that HCM is uniformly monogenic: recent studies show that common variant burden modifies penetrance and severity. Polygenic risk scores have been shown to stratify disease penetrance among pathogenic variant carriers by approximately tenfold and independently predict adverse outcomes, highlighting the potential future role of polygenic risk assessment for individualized family screening and risk stratification [31, 32].

Regarding family screening, the current guideline position is clear: all first-degree relatives should undergo either cascade genetic testing, if a (likely) pathogenic variant is known in the proband, or serial clinical screening [22, 23]. In G+ individuals without an established HCM phenotype, surveillance every 1–2 years in children and adolescents and every 3–5 years in adults is recommended, adjusted for symptoms, findings and family history. The intensity of long-term surveillance in G+ adults without HCM phenotype may eventually be individualized. In a 2025 cohort study of family screening, initial evaluation had high diagnostic yield, but only 6% of G+ individuals developed mild HCM over a median 8-year follow-up, with no adverse cardiac outcomes, supporting the idea that very low-frequency follow-up may be sufficient in selected low-risk adults after a thorough baseline evaluation [27].

Accumulating evidence indicates that G+ patients differ substantially from G- patients with regard to their risk profile [33, 34]. In a recent analysis of the international Sarcomeric Human Cardiomyopathy Registry (SHaRe) including 6120 patients, G+ patients were on average 16 years younger at diagnosis, and characterized by a higher risk for subsequent systolic dysfunction, AF and ventricular arrhythmias. G- patients, on the other hand, had a higher burden of modifiable risk factors such as arterial hypertension and obesity, and LVOTO. While all-cause mortality was similar, G+ patients were on average 7.8 years younger at their time of death and died more often of cardiovascular causes [33].

## Complication management

### Heart failure

**Fig. 2:**
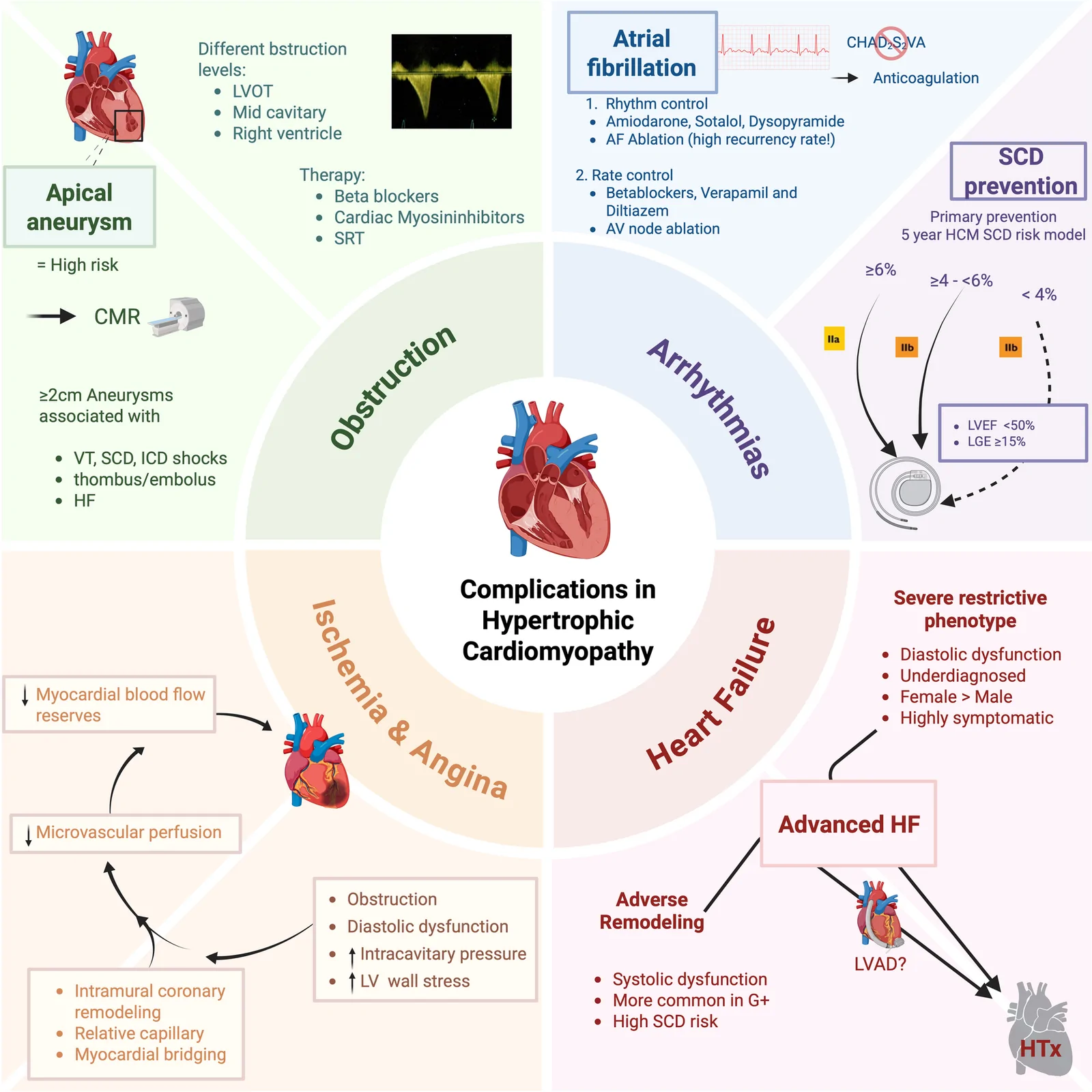
Complications in hypertrophic cardiomyopathy. AF, atrial fibrillation; AV, atrioventricular; CMR, Cardiovascular magnetic resonance; G+, genotype positive; HF, heart failure; HTx, heart transplantation; ICD, implantable cardioverter-defibrillator; LGE, late gadolinium enhancement; LV, left ventricle; LVAD, left ventricular assist device; LVEF, left ventricular ejection fraction; LVOT, left ventricular outflow tract; SCD, sudden cardiac death; SRT, septal reduction therapy; VT, ventricular tachycardia. Created in BioRender. Schwegel, N. (2026) https://BioRender.com/a800hu6

Heart Failure (HF) is a common complication in HCM. Especially in patients presenting at young age, up to 65% will experience HF symptoms during their lifetime [34, 35]. Over time, 3-8.5% experience progressive adverse remodelling, leading to advanced HF, commonly referred to as end-stage or burnt-out HCM [18, 36, 37]. One hallmark of HF in HCM is low cardiac output, driving symptomatic burden in affected patients. HF symptoms are also common in obstructive HCM. Dynamic LVOTO, often accompanied by mitral regurgitation, and impaired diastolic function contribute to elevated filling pressures and reduced exercise capacity [23, 38]. Although end-stage HCM with low output can present across a spectrum of phenotypes, two main morpho-functional presentations are commonly described:

(I) HCM with LV systolic dysfunction is characterized by excessive adverse remodelling, presenting with a LVEF < 50%. Dilative remodelling with a decrease in wall thickness and increase in LV diameter can be observed, although these changes are not required for a diagnosis [18, 39]. In this phenotype, the presence of one or more (likely) pathogenic variants is more common [18, 40], and the symptom onset is more likely to be at a younger age [18, 39, 40].
(II) Restrictive HCM features significant diastolic dysfunction, with supporting echocardiographic findings, including markedly elevated filling pressures and left atrial enlargement [41]. Importantly, diastolic dysfunction is an ubiquitous finding in HCM and contributes to HF symptoms throughout the disease spectrum, even in the absence of restrictive filling physiology [38]. In restrictive HCM, LV size is typically small to normal and systolic function is ≥ 50%. This phenotype commonly presents in an older population, mainly female, and with a higher symptomatic burden (NYHA class III/IV) [36] (Fig. 2).

#### Treatment

In HCM, HF treatment is an individualized approach based on phenotype and tolerability.

In obstructive HCM (oHCM) symptoms are primarily driven by dynamic LVOTO, leading to increased intracavitary pressures, reduced cardiac output, and mitral valve abnormalities. In contrast in non-obstructive HCM (nHCM) symptoms are frequently related to elevated LV filling pressures, impaired relaxation, and reduced chamber compliance and arise more from the intrinsic myocardial phenotype: myocyte disarray, fibrosis, diastolic dysfunction, microvascular ischemia, and electromechanical remodelling, than dynamic features like in oHCM [1, 42].

In oHCM cardiac myosin inhibitors have been established to directly target sarcomere-driven hypercontractility through reducing actin-myosin cross-bridge formation. Trials demonstrated functional and symptomatic improvements on top of beta blockers or calcium channel blockers including improved exercise capacity, symptomatic burden, patient-reported quality of life, LVOTO, and diastolic function [43–45]. The MAPLE-HCM trial also showed that myosin inhibitors were superior to standalone beta blocker therapy [46]. Myosin inhibition is expected to provide beneficial structural remodelling and prevent deterioration of the underlying disease, which could lead to the need for reevaluation of previously investigated treatments and risk stratification systems. As a disease-modifying treatment, this drug-class promises to delay or even prevent the transition to an advanced disease stage. However, long-term data are still needed. Further, cardiac myosin inhibitors are contraindicated in HCM with LVEF < 55%. In symptomatic oHCM with clinically relevant obstruction despite maximally tolerated medical therapy, septal reduction therapy (SRT) —surgical myectomy or alcohol septal ablation in experienced centres—should be considered when cardiac myosin inhibition is contraindicated, not tolerated, unavailable, or insufficiently effective [22]. Importantly, SRT remains a durable and effective strategy to abolish LVOTO and improve survival, and remains an important alternative strategy [47]. Of note, SRT should preferably be performed in experienced HCM centers, as outcomes are closely related to institutional and operator experience [48, 49].This is particularly relevant in the era of cardiac myosin inhibitors, which will reduce SRT referral volumes and thereby further concentrate expertise in dedicated centers.

Symptomatic nHCM has also become a logical target for cardiac myosin inhibition trials. Earlier, the phase II MAVERICK-HCM trial demonstrated favourable effects on cardiac biomarkers and established the safety of mavacamten in symptomatic nHCM [50]. In REDWOOD-HCM Cohort 4, aficamten in symptomatic nHCM was associated with improvements in symptoms and biomarkers [51], while the following ACACIA-HCM phase III trial has announced positive primary endpoint results for both, the Kansas City Cardiomyopathy Questionnaire (KCCQ) and peak oxygen consumption (peak VO₂) [52, 53]. However, the neutral ODYSSEY-HCM trial, in which mavacamten did not significantly improve peak VO₂ or health status despite biomarker effects, contrasted with the positive findings of ACACIA-HCM and underscores the biological heterogeneity of symptomatic nHCM, suggesting that differences in patient selection, trial design, endpoint selection, and underlying disease biology may influence treatment response and warrant further studies to identify patients most likely to benefit from myosin inhibition [54]. Beyond HCM, the EMBARK-HFpEF trial recently extended the evaluation of myosin inhibition to patients with HF with preserved and supranormal ejection fraction. Here, mavacamten was associated with significant reduction in cardiac biomarkers and improvements in exploratory measures of functional status and diastolic function, supporting the importance of patient phenotyping for myosin inhibition treatment [55]. Another therapeutic target in nHCM could be ninerafaxstat targeting more efficient glucose utilization through fatty acid oxidation inhibition, which is under investigation in ongoing Phase II trials [56, 57].

Randomized controlled trial evidence for anti-neurohumoral therapy in HCM remains limited. In symptomatic HCM patients, use of beta blockers for reducing filling pressures are recommended, despite limited randomized evidence supporting this approach, and should be individualized given the potential for chronotropic limitation, particularly in physically active patients [23, 42]. For younger G+ patients in an early disease stage the VANISH-trial favors valsartan for improving LV structure, function and biomarkers [58], though there is no recommendation by guidelines and evidence remain limited. Ongoing studies, including SONATA-HCM and EMPA-REPAIR, are evaluating the effects of sodium-glucose-cotransporter (SGLT) 1 and SGLT 2 inhibition on disease progression and clinical outcomes in HCM [59, 60].

Significant (i.e. at least moderate) mitral regurgitation (MR) is a common finding in HCM, with reported prevalences of 36% in oHCM, 22% in end-stage HCM, and 9% in other forms of HCM [61]. In both oHCM and end-stage HCM, significant MR is not independently associated with adverse outcomes [61]. In oHCM, it is in the majority due to systolic anterior motion (SAM) and LVOTO and can be relieved with successful treatment of LVOTO in most cases [62, 63]. In fact, the presence of significant MR is indicative of septal reduction therapy in patients with oHCM and NYHA functional class II [22]. Intrinsic mitral valve defects warranting surgery occur in approximately 10% of oHCM patients [63]. Here, surgical myectomy with mitral valve repair should be preferred over surgical myectomy with mitral valve replacement, due to better early and long-term outcomes [64]. In nHCM, at least moderate MR carries prognostic significance. Both in end-stage HCM and nHCM with LVEF ≥ 50%, guideline-recommended criteria for MR quantification and treatment apply [65].

#### Advanced heart failure

Advanced HF in HCM is generally characterized by persistent severe symptoms, usually NYHA class III–IV despite guideline-directed therapy, with or without reduced LVEF [23].

End-stage (burn-out) HCM is characterized by adverse ventricular remodelling and was previously reported at 14 ± 10 years from initial diagnosis, leading to death or heart transplant (HTx) within 2.7 ± 2 years following an end-stage diagnosis. While SCD risk in patients with LV systolic dysfunction is about fivefold higher compared to patients with normal LV function, ICDs have markedly improved survival, shifting the primary cause of mortality towards HF, accounting for about 50% of deaths in HCM [14, 66]. Through considerably improved management and evaluation strategies, contemporary outcomes in end-stage HCM show significantly improved survival, with a 10-year survival rate of 83% and a mortality of 2.2% annually [40]. Analyses from the SHaRe indicate a time from diagnosis of LV systolic dysfunction to a combined endpoint of all-cause mortality, HTx, or implantation of a left ventricular assist device (LVAD) from up to 8.4 years. Nevertheless, these events remain prevalent, still affecting approximately 35% of patients with end-stage HCM [18]. While literature is mostly focused on HCM with LV systolic dysfunction, patients may also present with advanced HF symptoms in the presence of preserved LVEF. This phenotype, mostly with restrictive physiology, affects about 6% of HCM patients and remains underdiagnosed and undertreated, although HCM-related mortality or the requirement for advanced treatment is comparable to HCM with LV systolic dysfunction (Fig. 2). Progressive restrictive physiology with preserved LVEF is typically characterized by worsening filling pressures, marked left atrial remodelling, increasing myocardial fibrosis, and persistently small ventricular cavity size rather than LV dilatation or systolic impairment [36, 67].

If LVEF is below 50%, neurohumoral modulation with beta blockers, angiotensin-converting enzyme (ACE) inhibitors, angiotensin receptor blockers (ARBs), mineralocorticoid receptor antagonists (MRAs), SGLT 2 inhibitors and angiotensin-receptor-neprilysin-inhibitors (ARNIs), the standard therapeutic approach in HF with reduced ejection fraction, is recommended empirically (Class IIa/IIb indication) [23], even though specific evidence in HCM is lacking. Cardiac resynchronization therapy should also be considered in the setting of left bundle branch block (LBBB), LVEF < 50% and symptoms despite optimal medical therapy. Advanced HF strategies, especially the use of LVAD, are limited in HCM due to small cavity size and severe LVH. These technical and physiological limitations made LVAD implantation in HCM relatively uncommon. However, in selected patient it might be considered as a bridge-to-transplant strategy, with or without concomitant myectomy [68, 69]. Studies demonstrated that when successfully implanted, survival and length of hospital stay are comparable to those in patients with ischemic or dilated cardiomyopathy [70, 71]. Yet, especially patients with restrictive phenotypes have a high risk of perioperative complications, therefore upfront HTx is preferred over a bridge strategy with LVAD [72]. The timing of HTx remains challenging especially in the younger population. Functional capacity commonly is overestimated by treating physicians and traditional peak VO₂ thresholds for transplantation (12 or 14 mL/kg/min, depending on established beta-blocker therapy) may underestimate risk especially in young HCM patients. Common markers of poor prognosis are LVEF < 50%, severe restrictive phenotype, presence of overtly small ventricular cavities, decrease in peak VO₂ on cardiopulmonary exercise testing < 50% of expected values, and refractory lift-threatening arrhythmias which should be considered indicators for HTx [23, 73]. Post-transplant and longer-term outcomes in HCM are overall excellent: 1-year survival in HCM is over 90%, and 5-year survival (83%) surpasses both ischemic and non-ischemic cardiomyopathy survival outcomes (75% and 77%, respectively) [23, 74]. Of note, recurrence of HCM in the transplanted heart was not observed in histological evaluation [75]. These data support the current class I recommendation for HTx in HCM patients with advanced HF refractory to treatment [22, 23].

### Cavitary obstruction and apical aneurysm

LVOT obstruction in HCM is caused by systolic anterior motion of the mitral valve and septal hypertrophy, leading to dynamic pressure gradients, increased intracavitary pressures, and reduced cardiac output. While this form represents the most common and well-characterized form of obstruction [15], similar hemodynamic principles also apply to less recognized phenotypes such as cavitary obstruction, which represents an important distinct HCM phenotype, associated with the development of apical aneurysm and an increased risk of adverse outcomes. Cavitary obstruction is defined as LVH that causes obstruction in other ventricular regions than the LVOT. This obstruction can progress to the point where it has haemodynamic consequences, causing increased pressure gradients throughout the LV cavity, ultimately leading to apical aneurysm formation. With progressive hypertrophy, this can ultimately even lead to functional separation of the basal and apical compartments, causing detrimental intra-cavitary pressure rises.

Beyond its obvious association with apical aneurysm, mid-ventricular/cavitary obstruction (MCO) is considered a high-risk phenotype linked to a higher degree of symptoms, increased risk of arrhythmias and progression to end-stage HF [76–78]. MCO is also associated with more ventricular arrhythmias and ICD shocks [79, 80], with increasing intraventricular pressure gradients (especially ≥ 30 mmHg) associated with a higher likelihood of appropriate ICD therapy and SCD [81].

Treatment data for symptomatic MCO are limited and derived from observational cohorts. Medicinal approaches (high dose beta blockers, diltiazem and verapamil) are recommended by the current ESC guidelines to decrease heart rate and intracavitary pressures, but often fail to reach a clear response.

Anecdotal case reports have suggested a benefit in terms of gradient and biomarker reduction in patients with mid-cavitary obstruction treated with the cardiac myosin inhibitor mavacamten [82, 83]. An exploratory subgroup analysis of REDWOOD-HCM Cohort 4 showed that 7 patients with MCO and nHCM showed symptom improvement and reductions in biomarkers after treatment with aficamten [51, 84]. Subgroup analyses of ODYSSEY-HCM included the variable of MCO (< 30mmHg vs. ≥ 30–50 mmHg) and did not show a clear difference on the primary endpoints of KCCQ and peak VO_2_. Interestingly, a placebo-controlled trial on mavacamten in mid-cavitary (non-LVOT) obstruction is currently enrolling. Together with subset analyses of other trials (e.g. ACACIA-HCM), this will provide more definitive evidence on the potential role of myosin inhibition in this particular subgroup [53, 85].

Surgery (either transapical, transaortic or combined transaortic myectomy procedures) has been successful in the hands of experienced high-volume centers, with short-term outcomes of these procedures showing good responses on symptoms and gradient reduction, although long-term outcomes and survival are still unclear [86]. Its use and success outside of these high-volume centers remains less clear.

Apical aneurysm is defined as a thin-walled akinetic or dyskinetic apical segment, which in the setting of HCM is most commonly caused by obliterating cavitary obstruction. This is often accompanied by extensive apical scar formation, which can be visualized by CMR. Apical aneurysms are a substrate for malignant arrhythmias and can also result in thromboembolism. Cohort studies suggest a prevalence of approximately ± 3–5% of apical aneurysms in HCM patients [87, 88], with up to 28% in HCM patients with mid-ventricular obstruction [76, 77]. Importantly, the use of CMR imaging has likely increased the detection of apical aneurysms, as echocardiography is sometimes unable to detect small aneurysms. The clinical consequences of these small aneurysms remain to be further studied, as large aneurysms carry greater risks and are more likely to be detected. The size matters, with a commonly used cut-off of 2 cm as the threshold for increased risk for thrombus and stroke and potentially also arrhythmias and SCD [89, 90] (Fig. 2). Apical aneurysms should be assessed using contrast-enhanced echocardiography and, when suspected or incompletely visualized, confirmed by CMR, which provides superior detection of small aneurysms and more reproducible measurements of the maximum transverse aneurysm diameter. Conventionally, aneurysms have been classified as small (< 2 cm), moderate (2–4 cm), and large (> 4 cm), although recent data suggest that risk increases continuously with aneurysm size [87, 89]. A recent largest-to-date analysis of 510 HCM patients with LV aneurysms confirmed this finding, suggesting a linear relationship between aneurysm size and risk, with increased risks above the threshold of 1 cm [91].The current ESC and AHA guidelines recognize apical aneurysms as an important distinct risk factor, with the AHA guidelines also using apical aneurysms in general (regardless of size) as a major risk factor in their SCD risk assessment [22, 23]. Importantly, the AHA guideline definition is ‘a discrete thin-walled dyskinetic or akinetic segment with transmural scar or late gadolinium enhancement (LGE) of the most distal portion of the LV chamber, independent of size´. The ESC guideline does not incorporate apical aneurysms as a standalone major risk factor (nor has a specific definition for it), and defines SCD risk on the HCM-SCD Risk score models.

Currently, there is no strong evidence to support routine prophylactic anticoagulation for HCM-related apical aneurysms in the absence of atrial fibrillation, prior thromboembolism, or documented LV thrombus. The 2023 ESC cardiomyopathy guideline nevertheless states that anticoagulation may be considered in selected patients, based on observational data suggesting increased thromboembolic risk [22, 87]. The mentioned recent analysis of 510 patients with HCM and apical aneurysms showed apical thrombus or thrombo-embolic events in respectively 13% of the cases, again with a linear relationship with LV aneurysm size, highlighting the need for individualized decision-making in this setting, while also taking into account other factors like atrial fibrillation, LVEF and concomitant bleeding risks [91]. When an apical thrombus is demonstrated, oral anticoagulation is generally indicated. However, an HCM apical aneurysm differs from transient LV dysfunction because the akinetic aneurysmal substrate usually persists. Therefore, after thrombus resolution, long-term or indefinite anticoagulation may reasonably be considered [89].

If ventricular arrhythmias occur from apical scar regions, anti-arrhythmic drugs and ventricular tachycardia (VT) ablation are therapy choices. In rare, therapy-refractory ventricular arrhythmias, aneurysmectomy can be considered in experienced centers [90, 92].

### Atrial fibrillation

AF is the most prevalent arrhythmia in HCM, identified in 20–25% of patients during their lifetime. The incidence is 2–4% per year and significantly higher compared to an age and gender matched population [16, 17, 93]. AF in patients with HCM is associated with an increased symptom burden, stroke risk, HF and hospitalization [93, 94].

The increased risk to develop AF in HCM is caused by several disease-specific characteristics; atrial dilatation, fibrosis and (contractile) dysfunction and underlying ion-channel abnormalities all lead to an atrium prone for AF development [22, 95, 96]. Underlying pathogenic sarcomeric variants are found in patients presenting with early-onset AF, without a HCM phenotype suggesting that the primary genetic cause also increases the risk for AF development [95]. This is further supported by a twofold higher risk in G+ compared to G- patients with HCM [97].

AF is generally poorly tolerated in patients with HCM due to a decline in diastolic ventricular filling caused by the loss of atrial contraction and the shortening of diastolic time associated with faster heart rates. It leads to (worsening) HF symptoms with exercise intolerance caused by dyspnea and chest pain and fluid retention.

The treatment of AF in HCM patients primarily focuses on the prevention of embolic events and the restoration of sinus rhythm. Patients with HCM and AF have an increased risk for embolic events compared to patients without HCM and AF. The commonly used CHA_2_DS_2_-VA risk score underestimates the risk of embolic stroke in patients with HCM and international guidelines therefore recommend anticoagulation for clinically overt AF irrespective of this score [22, 98].

Rhythm control in HCM patients is challenging and evidence to guide therapeutic choices is lacking, as the use of antiarrhythmic drugs and catheter ablation have not been studied in randomized controlled HCM trials [22, 96]. Current ESC guidelines support the use of disopyramide, sotalol and amiodarone for rhythm control [22]. Disopyramide is not widely available and has the disadvantage of anticholinergic side effects. Sotalol should be used with caution as evidence suggests that it might be associated with increased mortality [99]. Amiodarone is considered most effective for rhythm control in HCM but has side-effects, especially during long-term use. Catheter ablation for AF in patients with HCM reduces the AF burden, but the rate of AF recurrence after ablation is substantial and the need for repeat ablation is higher in patients with HCM compared to patients without HCM [100]. If rhythm control fails, rate control should be pursued. Guidelines support the use of beta blockers, verapamil and diltiazem [22]. Atrioventricular (AV) node ablation is an option if all other therapeutic strategies fail to control the heart rate in AF in patients with HCM [101] (Fig. 2).

### Ischemia and angina

Ischemia and angina are common phenomena in HCM, often without significant concomitant coronary artery disease. Next to dyspnoea and exercise limitation, chest pain is one of the most frequently reported symptoms, with some studies suggesting up to ± 70% of symptomatic patients experiencing some form of angina and up to ± 20% of HCM patients experience anginal symptoms in all-comer HCM populations [102, 103].

Already in 1986, a necropsy study showed ‘small vessel coronary disease’ in deceased HCM patients, compared to non-HCM patients with decreases in luminal size and increased wall thickening [104]. These observations are in line with the observations of intramural coronary remodelling, extravascular compression, relative capillary decrease compared to myocyte mass and increased oxygen demands in HCM, all contributing to an oxygen supply-demand mismatch in HCM patients [105]. Nuclear perfusion and CMR studies have consistently shown the presence of perfusion abnormalities and impaired myocardial blood flow reserves in HCM patients [106, 107]. The degree of microvascular dysfunction measured before and after dipyridamole with positron-emission tomography has clearly shown an association with clinical outcomes, also in the absence of symptoms [108], with many more later studies confirming this observation. More recent studies using angiography-derived resistance measures also showed increased resistance measures in HCM patients compared to controls, again associated with clinical outcomes [109].

Although microvascular dysfunction is one of the obvious mechanisms of angina in HCM, perfusion abnormalities are clearly not synonymous to angina occurrence. Many non-invasive studies to date have shown no clear relationship with perfusion defects and the presence of symptoms or reduced exercise duration, suggesting that anginal symptoms are not only explained by (the extent of) ischemia, and overt ischemia can be present without any symptoms [110, 111]. Most likely, ischemia explains the symptom burden only partly, with additional contributors likely including dynamic LVOTO or MCO, diastolic dysfunction, elevated intracavitary pressures and increased LV wall stress. Concomitant epicardial coronary artery disease also can contribute to a potential ischemic component, and is associated with a reduced prognosis in HCM patients [112]. Myocardial bridging is also considered a potential reason for myocardial ischemia and subsequent angina (Fig. 2). Myocardial bridging is defined by the compression of epicardial coronary arteries during myocardial contraction, resulting in impaired blood flow. Current data on the association between myocardial bridging and angina are conflicting, with a recent study showing that the presence of myocardial bridging on angiography was associated with angina [113], while another study showed that angina in HCM patients is not associated with the presence of myocardial bridging on coronary computed tomography scans [114]. A recent meta-analysis shows a relationship of bridging with ischemia, and the presence of myocardial bridging does translate into worse clinical outcomes [115].

Treatment of angina is specifically addressed in the current ESC guideline, with the consideration of beta blockers or calcium antagonists as first-line therapy in patients with typical angina-like symptoms, even in the absence of an LVOT gradient or significant coronary artery disease. However, this advice is based only on small studies. Oral nitrates can be used cautiously in selected patients, although one should be careful in oHCM patients. Ranolazine can also be considered in nHCM patients for therapy-refractory angina according to the ESC guidelines, with evidence from experienced centers showing it to be safe and successful [116]. However, it failed to show any overall functional benefits in the RESTYLE-HCM trial and is also not available in all health care systems [117]. Interestingly, the myosin inhibitor aficamten has also shown marked improvements on the Seattle Angina Questionnaire Summary Score (SAQ-SS) assessments in the SEQUOIA-HCM trial compared to placebo [118], and the MAPLE-HCM trial [119] in oHCM. The REDWOOD-HCM Trial Cohort 4 also showed a significant improvement in SAQ-SS in 13 nHCM patients treated with aficamten [51].

### Sudden cardiac death

SCD was one of the leading causes of death in HCM, especially among younger patients. Since the introduction of ICDs the annual HCM-related mortality declined to approximately 0.5% [120]. According to the current ESC guidelines ICD implantation for secondary prevention is clearly indicated in SCD survivors caused by VT or ventricular fibrillation, as well as in those with sustained haemodynamic relevant VT [22]. In contrast, risk stratification for primary prevention ICD implantation is more challenging as patients at the highest risk for ventricular arrhythmias need to be identified, while unnecessary ICD implantations should be avoided to minimize device-related complications such as infections, malfunction, or inappropriate ICD therapy. A recent review by Kasiakogias et al. provides a comprehensive overview of the current challenges surrounding SCD risk stratification [121].

#### Primary prevention and risk models

Various models to estimate SCD risk in HCM patients have been recommended over the last decades by the ESC and AHA [22, 23, 122]. In 2014 O´Mahony et al. presented the HCM Risk-SCD model incorporating seven SCD predictors as age, family history of SCD, maximum wall thickness (MWTH), left atrial diameter, maximal LVOT gradients, presence of non-sustained VT, and a history of unexplained syncope [123]. The ESC CMP guidelines recommend ICD implantation as a class IIa indication in high risk patients with a calculated 5-year SCD risk of ≥ 6% and as a class IIb indication in patients with an intermediate risk of ≥ 4% to < 6%. In patients with an estimated low SCD risk (< 4%) ICD implantation should be considered as a IIb indication by assessing additional parameters as LVEF < 50% or presence of extensive LGE > 15% [22, 122]. (Fig. 2). In contrast, according to the AHA HCM clinical practice guidelines all patients with at least one major risk factor for SCD including a positive family history for SCD, massive LVH ≥ 30 mm, a prior syncope, apical aneurysm or LVEF < 50% should be evaluated for ICD implantation as class IIa indication [23]. While the AHA guidelines consider the presence of apical aneurysm as a major risk factor for SCD, the ESC guidelines follow a more cautious approach, recognizing it only as an additional risk modifier due to the limited data currently available [22, 23]. A recent analysis from the SHaRe compared ICD implantation rates and outcomes between centers of the United States of America (USA) and non-USA centers showing higher ICD implantation rates in the USA with lower rates of adequate ICD therapies. Interestingly there was no significant difference in outcomes in patients who did not receive ICD implantation between USA and non-USA hospitals [124]. This demonstrates that indications for primary prevention ICD implantation vary across countries, and it highlights the need for refinement of current SCD risk-prediction models to improve patient selection. Data on the additional value of SCD risk prediction in patients with LGE but without extensive involvement (< 15%) remain limited and conflicting. A recent study by Wang et al. investigating this association suggests that incorporating LGE within the range of 5–15% involvement may provide incremental value as an additional risk marker for SCD [125]. This finding is supported by the meta-analysis by Kiaos et al., which identified an LGE threshold of 10% as the optimal cutoff for SCD risk stratification, while highlighting it as an additional risk marker rather than an independent predictor [126]. Emerging therapies such as cardiac myosin inhibitors reduce LVOT gradients and might even promote cardiac remodelling by reducing MWTH and left atrial size leading to a consequently lower calculated SCD risk. However, whether this translates into a real reduction in SCD incidence remains to be determined in long-term follow-up. While ICD therapy has markedly reduced SCD-related mortality in patients with HCM and various SCD risk prediction models already exist, a uniform recommendation is still lacking and further studies to identify patients at the highest risk are still warranted.

## Conclusion

Complication management is central to improving outcomes in HCM, as morbidity and mortality are largely driven by dynamic obstruction, atrial fibrillation, ischemia, ventricular arrhythmias, and progression to advanced HF. Contemporary care therefore requires systematic surveillance for these complications and timely escalation to phenotype-specific therapies, including SRT for refractory obstruction, anticoagulation and rhythm-control strategies for AF, ICD implantation in selected patients at increased SCD risk, and early referral to advanced HF centers when restrictive physiology or systolic dysfunction emerges.

Importantly, HCM complications frequently overlap and amplify each other. LVOT or mid-cavitary obstruction may promote HF symptoms, mitral regurgitation, ischemia, and apical aneurysm formation; AF may precipitate decompensation in patients dependent on atrial contribution to ventricular filling; and fibrosis, aneurysms, or systolic impairment may increase arrhythmic risk. Management should therefore not rely on isolated findings but integrate symptoms, hemodynamics, imaging, genotype, rhythm burden, and longitudinal disease trajectory.

Emerging therapies, particularly cardiac myosin inhibitors, are expanding the treatment landscape and may alter complication pathways by reducing obstruction and potentially modifying adverse remodelling. However, their long-term impact on HF progression, AF, SCD, and survival remains to be defined. Future studies must refine risk prediction and identify which patients benefit from earlier, targeted intervention before irreversible complications develop.

## Data Availability

No datasets were generated or analysed during the current study.
